# Adapting to the digital age in psychiatry: evaluating change in emergency department nurses and psychiatrists' views toward telepsychiatry for involuntary hospitalization

**DOI:** 10.3389/fdgth.2026.1810008

**Published:** 2026-05-12

**Authors:** L. Shalev, G. Lubin, S. Kirresh, I. Barash, A. Tamary Guterman, E. Bloemhof-Bris, S. Hirschmann, A. Avni, K. Avirame, M. Linder, D. Bathish, R. Badran, Y. Turm, B. Bloch, S. Konas, L. Izakson, F. Asad, Y. Melamed, S. Raskin, D. Raz, R. Eitan, A.J. Rose

**Affiliations:** 1 Braun School of Public Health and Community Medicine, Hebrew University of Jerusalem, Jerusalem, Israel; 2 The Jerusalem Mental Health Center, Jerusalem, Israel; 3 Lev Hasharon Mental Health Center, Tzur Moshe, Israel; 4 Psychiatric Division, Sourasky Medical Center, Tel Aviv-Yafo, Israel; 5 Faculty of Medical & Health Sciences, Tel-Aviv University, Tel Aviv-Yafo, Israel; 6 Sha'ar Menashe Mental Health Center, Shaar Menashe, Israel; 7 The Ruth and Bruce Rappaport Faculty of Medicine, Department of Psychiatry, Technion, Haifa, Israel; 8 Psychiatric Department, Emek Medical Center, Afula, Israel; 9 Geha Mental Health Center, Petach Tiqwa, Israel; 10 Psychiatric Division, Ziv Medical Center, Safed, Israel; 11 Azrieli Faculty of Medicine, Bar-Ilan University, Safed, Israel; 12 Abarbanel Mental Health Center, Bat Yam, Israel; 13 Ministry of Health, Jerusalem, Israel; 14 Ministry of Justice, Jerusalem, Israel

**Keywords:** adaptation, digital health (eHealth), emergency department, implementation science, innovation, organizational change, telepsychiatry

## Abstract

**Introduction:**

Implementing change in organizations is challenging, and a key factor in success is the perception of the implementers. While many studies report on implementers' perceptions as barriers or facilitators for implementing innovations, they often do not examine how these perceptions change over time. We aimed to evaluate changes in perceptions among nurses and psychiatrists in emergency departments (EDs) regarding the implementation of telepsychiatry (live video) for involuntary hospitalization.

**Methods:**

This study utilized quantitative and qualitative methods, administering an online questionnaire to nurses and psychiatrists in eight EDs in Israel before and after telepsychiatry implementation. The questionnaire included: 1. Background characteristics (i.e., role and seniority); 2. SHEMESH questionnaire (“Organizational Readiness to Change Assessment”) comprising 11 items about the evidence for telepsychiatry, its feasibility, its effectiveness compared to face-to-face evaluation, and items about how supportive the ED environment for implementing changes in practice; 3. Two open-ended questions about how telepsychiatry would fit patients and the challenges in using telepsychiatry for involuntary commitment. Quantitative data were analyzed using descriptive statistics, *t*-tests, and regression models, while qualitative data were analyzed and categorized.

**Results:**

344 participants completed the questionnaire (54% from the pre-implementation phase and 46% from the telepsychiatry phase). Both phases showed high scores on the overall Evidence construct, indicating that telepsychiatry is evidence-based, with an increase from the pre-implementation to the telepsychiatry phase. High scores on the overall Context construct were also observed, with no difference between phases. A regression model identified study phase, psychiatrists, and management team as predictors of higher Evidence and Context scores. Respondents' comments in the pre-implementation phase focused on concerns about feasibility, while comments in the telepsychiatry phase focused on patient cooperation and agitated patients. Questions about professionalism arose in both phases. Overall, negative responses decreased significantly during the telepsychiatry phase, while perceptions of telepsychiatry's fit for patients increased, though not statistically significantly.

**Discussion:**

Ongoing use of an innovation over time can result in a favorable change in implementers' perceptions as they grow accustomed to the innovation and appreciate its advantages, such as increased efficiency and improved results.

**Clinical Trial registration:**

ClinicalTrials.gov identifier: NCT05771545, March 15, 2023.

## 1 Introduction

Implementing change and innovation in organizations is a challenging task. One of the key factors in the success or failure of implementation is the perception of the implementers (1). Implementers play a critical role in successful implementation, particularly in settings where improved patient quality of care is essential, such as in emergency settings (2, 3). While many studies report on implementers' perceptions in emergency settings as barriers or facilitators for implementing innovations, studies seldom examine of how these perceptions change over time (4–6). In one study, Spitler and colleagues (7) evaluated the impact of 24/7 attending neuroradiology coverage on radiology residents' on-call experience, referring physicians' satisfaction, and report turnaround times at a large academic medical center. The results showed improved report accuracy, timeliness, and accessibility for referring physicians, while radiology residents reported enhanced educational value and independence (7). This study examined changes in perceptions, but only after implementing the change. Another study by Voutsinas and colleagues (8) aimed to improve communication between the ED and radiology by developing a web-based tool to reduce misdirected phone calls, which were causing delays and interruptions in care. After implementing the tool, misdirected calls significantly decreased, and over 80% of ED respondents adopted the tool, leading to improved efficiency and accuracy in communication, with plans to expand its use hospital-wide (8). However, this study focused on practice improvement but did not report on the perceptions of front-line staff before or after the change. Implementing change and innovation is also challenging in psychiatric emergency settings.

Psychiatric EDs can be an intensive settings due to the nature of the arriving patients, who might experience delusional thoughts, hallucinations, agitation, and violent outbursts (9–11). This creates a challenging and potentially dangerous environment for both the patients, other patients and staff members (12). Prolonged waiting time can exacerbate patients' mental state, often necessitating sedation or physical restraint to prevent harm (13). The lack of proper evaluation rooms and external stimulation can contribute to further deterioration in patients' conditions (14). These circumstances can contribute to staff burnout (15, 16). All those factors can significantly compromise the quality of patient care, including clinical decision-making (17). Over the years, innovations have been implemented in the ED to cope with those challenges.

Over time, several solutions have been applied in the psychiatric ED with the goal of improving patients' care. Efforts to address patient agitation in the ED have included pharmacological innovations tailored to individual patient needs (18). Additionally, tools have been developed not only to recognize agitation, but also to manage its underlying causes, ensuring thorough and effective care (19). Artificial intelligence solutions, such as machine learning techniques, have been applied in psychiatric EDs to enhance clinical decision-making and offer new insights (20). Further improvements include community-based mobile programs that divert less acute patients from EDs to more appropriate care settings (21). Other interventions focused on addressing the gap between EDs and community resources and creating innovative pathways to better meet psychiatric patients' needs (22). Another innovative solution in the ED is implementing telepsychiatry, a video meeting between a psychiatrist and a patient, for psychiatric emergencies (23).

Telepsychiatry has seen increasing use over the years for evaluation, treatment, and follow-up (24). Telepsychiatry evaluation in the ED has been shown to be valid compared to in-person psychiatric evaluation (25, 26). Telepsychiatry has potential to reduce the time psychiatric patients spend in the ED (27, 28). By providing expedited evaluations and treatments, telepsychiatry can prevent further mental deterioration in patients (29, 30). Telepsychiatry can also contribute to decreased organizational costs (28, 31). Despite its documented growing use in the United States (32, 33), there is still limited understanding of the barriers and facilitators involved in implementing telepsychiatry in EDs (34).‏

Over the past three decades, it has become clear that developing effective treatments is not enough (35), and that there is a need to ensure their adoption and sustainability (36). This realization has led to the emergence of Implementation Science, a field focused on creating reproducible methods to facilitate the uniform adoption of proven clinical practices and addressing barriers to their implementation (3). The growth of Implementation Science underscores the need to better understand and implement innovations like telepsychiatry in health care settings (37).

When a change or innovation is introduced in a new setting, understanding enablers and barriers to the change is crucial to determining why interventions succeed or fail (38, 39). This is important in healthcare settings, especially in EDs, where improving quality of care with limited resources is vital (37). One of the key factors affecting successful implementation is the context (40). Organizations and managers that foster cooperation, collaboration, and communication are more likely to succeed in implementing innovations and changes (41). Assessing staff perceptions in advance can help organizations measure the likelihood of successful implementation and, if needed, improve these contextual factors to better prepare for the change (36).

The aim of this study is to evaluate change in implementers views of telepsychiatry over time, comparing a period before telepsychiatry begins with a period after it is already in use. In this study, we present change in perceptions of nurses and psychiatrists working at the ED, about implementing telepsychiatry for involuntary hospitalization. This study focused on the degree of the strength of the evidence for telepsychiatry and the context factors, such as cooperation, collaboration, and communication. This study is guided by two Implementation Science frameworks. One conceptual framework is the Promoting Action on Research Implementation in Health Services framework (PARHIS) (42). The PARIHS framework suggests that successful implementation depends on three constructs: 1) Evidence-the perceived strength, feasibility, and relevance of the innovation; 2) Context-organizational factors that support or hinder implementation; and 3) Facilitation-efforts by researchers or clinical champions to promote the change. Successful implementation reflects how fully an innovation is adopted as standard practice. An additional conceptual and theoretical framework is the Consolidated Framework for Implementation Science Research (CFIR) (41). CFIR includes five domains: 1) Implementation Process-planning, executing, and continuously evaluating the innovation implementation; 2) Inner Setting-organizational characteristics such as hierarchy, culture, and resources invested in projects; 3) Innovation characteristics-perceptions of the innovation's evidence and advantages compared to the usual care; 4) Individual's characteristics-implementers and recipients' perceptions, beliefs, skills and abilities; and 5) Outer setting-environmental factors like geography, population, and relevant laws and regulations affecting implementation**.**

## 2 Materials and methods

This study is part of a larger study examining patient outcomes under telepsychiatry evaluation, compared with usual practice (23). The present analysis focuses on evaluating changes in implementers' views of innovations over time, specifically examining the implementation process of telepsychiatry in the ED setting, and how these views evolved over time. We investigated the perceptions of nurses and psychiatrists working in the ED regarding the use of telepsychiatry as part of the evaluation process to determining the need for involuntary psychiatric hospitalization using quantitative and qualitative methods.

### 2.1 Procedure

The current study is part of a larger project on implementing telepsychiatry in EDs in Israel. In Israel, involuntary psychiatric hospitalization can occur with a district psychiatrist's order for evaluation.‏ In contrast to patients who consent to be examined or hospitalized, patients who are considered for involuntary examination or hospitalization must be evaluated in person by an attending psychiatrist. For those patients, if the attending psychiatrist finds that the patient poses a risk to himself or others and does not consent to hospitalization, the case is presented by phone to the district psychiatrist, who can approve or decline the involuntary hospitalization (43).

In our study, the procedure was conducted similarly, except that during hours when attending psychiatrists are not ordinarily present (i.e., weekends, holidays, and from 3 PM-7 AM). During those periods, the attending psychiatrist's evaluation was performed via a secured video-link commonly used in healthcare settings (Datos software). The resident psychiatrist and the patients were located in the ED examination room using audio-visual equipment-either a laptop with speakers or a television screen with an attached camera, depending on local ED configurations, while the attending psychiatrist connected remotely from a separate computer (from home or elsewhere).

For this approach, we developed a structured protocol outlining the process for evaluating patients considered for involuntary admission. The protocol and the study rationale were introduced through structured training sessions at each participating hospital, conducted either face-to-face or remotely. Additionally, each hospital designated a lead attending psychiatrist and a resident psychiatrist who were responsible for providing in-person updates to the team on the procedure and serving as points of contact for questions and requests. One of the researchers (LS) was also available to all participating hospitals to address any additional inquiries. The protocol was distributed to all participating staff, and printed copies were made available in the ED examination rooms at each site.

The protocol and overall approach were approved by the Ministry of Health, which also granted a temporary waiver permitting remote evaluations during the study period (23).

A study conducted by our group in 2021 found high interrater agreement between telepsychiatry and in-person evaluations for involuntary admissions, supporting the reliability of remote evaluations. Additionally, psychiatrists and patients reported similarly high satisfaction levels across both modalities, and psychiatrists' perceived diagnostic certainty was high in both face-to-face and telepsychiatry evaluations (25). However, to the best of our knowledge, our large-scale study is the first to examine the effectiveness of telepsychiatry versus face-to-face evaluations in the context of involuntary admissions. Evidence in this area remains limited, and we are currently in the final stages of publishing our findings. Therefore, there is currently no robust evidence regarding the effectiveness of this approach at scale.

### 2.2 Setting

The study was conducted in eight EDs at seven hospitals in Israel. The participating hospitals volunteered to participate in the study and included three general hospitals and four psychiatric hospitals. Two hospitals are located in Israel's largest cities, Jerusalem and Tel Aviv, two in the northern periphery, and three in medium-sized cities. Of the eight ED's, two are affiliated with the same hospital in Jerusalem, but are located in different areas of the city.

### 2.3 Research phases

This study has two research phases: the pre-implementation phase and the telepsychiatry phase. In each hospital, a questionnaire was administered twice: two months before implementing telepsychiatry in the ED, and two months before completing the telepsychiatry phase. Each hospital joined the research in a different month during 2023–2024, with a research duration of 4–6 months. For the pre-implementation phase, the questionnaire was administered for the first time in May 2023 and in four hospitals in February 2024. For the telepsychiatry phase, the questionnaire was administered in May 2024, after each hospital had used telepsychiatry for at least three months.

In Israel, a war began on October 7th, 2024. Aspects of care delivery at hospitals, especially staffing, were extremely different from the usual pattern during the first several months of the war, due in part to a large call-up of many key staff to military reserve service (44). Therefore, we paused the study for four months and restarted it on February 1st, 2024. Therefore, questionnaires from the telepsychiatry phase were collected after this date, when ED staffing was mostly back to normal.

### 2.4 Participants

The present study involved two groups of participants. The first group included the ED staff-attending and resident psychiatrists and nurses working in the ED. The second group included the hospital management, the hospital head and heads of services and units from the medical and nursing teams.‏ Notably, psychiatry and nursing managers are also actively involved in clinical practice; they often participate in direct patient care, serve as senior decision-makers in complex cases, and function as on-call experts during shifts.

### 2.5 Data collection

We administered an online questionnaire that asked participants about the following:

1. Background characteristics-Hospital name, clinical team (medical/nursing), for psychiatrists, whether they are attending or resident psychiatrists, role in the hospital, and seniority in the ED (years).
2. The SHEMESH questionnaire (‘Organizational Readiness to Change Assessment’; In Hebrew: “SHE'elon Muchanut Ergunit le'SHinuy”) (45)-SHEMESH is the Hebrew-language version of the Organizational Readiness to Change Assessment (ORCA), a research tool developed based on the PARHIS framework (42). Participants rated their agreement from 1 (strongly disagree) to 5 (strongly agree) with 11 statements. The Evidence construct included four statements about the strength of the evidence, the feasibility of implementing psychiatric assessment via video-link at the ED, and the preference for this method compared to face-to-face evaluation. The Context construct included seven statements about the acceptability of quality improvement initiatives in one's department or unit, the decision-making process, and the communication and collaboration between medical and nursing teams in the ED. Originally, SHEMESH had high Cronbach's Alpha scores (Evidence: 0.887, Context: 0.852) (45). In the current study, Cronbach's Alpha scores were also high: 0.898 for the Evidence construct and 0.898 for the Context construct.
3. Perceptions about telepsychiatry-Two open-ended questions were developed for this study asking participants how they believe telepsychiatry will fit patients, and what challenges exist in using telepsychiatry in the ED for involuntary commitment.

Questionnaires with responses for fewer than half the items were excluded from the study.‏

### 2.6 Data analysis

All quantitative data were analyzed using SPSS (version 27.0). Descriptive statistics were used to describe the characteristics of the participants. Mean scores and standard deviations (SD) were calculated for all close-ended items. Items for the Evidence and Context constructs were aggregated into two overall scores, one for each construct. In the first step, independent-samples *t*-tests were conducted to examine bivariate differences between groups (e.g., psychiatrists vs. nurses or management vs. non-management roles). In the second step, multiple regression analyses with robust standard errors were conducted to predict perceptions of the overall Evidence and Context constructs. Robust standard errors were used due to non-normality of the dependent variables (i.e., Evidence and Context scores).

All qualitative data were analyzed through explanatory content analysis (46) based on the CFIR constructs (41). To achieve interrater reliability, two researchers (LS and AJR) validated the analysis to ensure the trustworthiness of the results. In case of disagreement, further discussions were held until agreement was reached.

### 2.7 Ethics approval and consent to participate

In accordance with the Declaration of Helsinki, approvals were from the research ethics committees of all involved hospitals: Emek Medical Center (EMC-130-23; 23 November, 2023); Geha Mental Health Center (23-23-GEH; 24 January, 2024); Jerusalem Mental Health Center (22-21; 6 November 2022); Lev-Hasharon Mental Health Center (LH12023; 12 February 2023); Tel-Aviv Medical Center (TLV-22-0656; 3 January 2023); Sha'ar Menashe (1-4-23; 18 April 2023); and Ziv Medical Center (74-23-ZIV, 8 November, 2023). Participants provided written informed consent to participate.

## 3 Results

### 3.1 Background variables

A total of 344 participants completed the questionnaire. Of them, 185 (54%) were from the pre-implementation phase and 159 (46%) were from the telepsychiatry phase. 257 (75%) of the respondents were attending or resident psychiatrists, and 87 (25%) were nurses. Most respondents (204, 60%) do not hold a managerial role at the hospital and work at the ED as psychiatrists or nurses, while 136 (40%) are in managerial positions, such as hospital director or head of a unit. The majority of respondents (313, 93%) currently work in the ED, and the rest (23, 7%) work only in management. No significant differences were found in background variables between the two research phases.

### 3.2 Perceptions about implementing telepsychiatry in the Ed

Participants were asked to rate their level of agreement on a scale of 1 (strongly disagree) to 5 (strongly agree) with 11 different statements across two constructs. The first construct, Evidence, includes four statements about the strength of the evidence for telepsychiatry, the feasibility of implementing telepsychiatry in the ED, and the preference for this method over face-to-face evaluation. High scores on the overall Evidence construct, indicating agreement that the intervention is evidence-based, was found in both phases. However, the overall Evidence score increased from the pre-implementation phase (M = 3.8, SD = 0.9) to the telepsychiatry phase (4.2, 1.0; *p* < 0.001) (Table 1).

**Table 1:** Mean (SD) score for sHEMESH questionnaire items for the evidence and context constructs by pre-implementation (*n* = 185) and telepsychiatry phases (*n* = 159; min = 1, max = 5).

Construct and items	Pre-implementation phase (*n* = 185)	telepsychiatry phase (*n* = 159)	*p*. value
**Overall Evidence construct**	**3.8** (**0.9)**	**4.2** (**1.0)**	***P*** **<** **0.001**
			***t*** **=** **−3.6**
1. Experts in your workplace agree with assessments via video-link	4.1 (1.1)	4.4 (1.1)	*p* = 0.03
			*t* = −2.2
2. Psychiatric evaluation via video-link will be feasible	3.9 (4.2)	4.2 (1.1)	*p* = 0.004
			*t* = −2.9
3. Psychiatric evaluation via video-link in the ED will be completed successfully	3.7 (1.0)	4.1 (1.2)	*p* = 0.001
			*t* = −3.2
4. There will be more advantages than disadvantages for patients from conducting psychiatric evaluation via video-link	3.5 (1.2)	4.0 (1.1)	*p* < 0.001
			*t* = −4.1
**Overall Context construct**	**4.0** (**0.7)**	**4.1** (**0.7)**	***p*** **=** **0.10**
1. Senior staff in the psychiatric ED encourage innovation and creativity with a goal of improving patient care	4.1 (0.9)	4.2 (1.0)	*p* = 0.40
2. Senior staff in the psychiatric ED encourages other members of the medical and nursing staff to share their opinions about how best to deliver care	3.9 (1.0)	4.1 (0.9)	*p* = 0.09
3. Senior staff in the psychiatric ED encourages improved approaches to delivering clinical care	4.1 (0.9)	4.1 (0.9)	*p* = 0.75
4. Senior staff in the psychiatric ED encourages teamwork between the medical and nursing staff to find solutions to improve patient care	4.0 (0.9)	4.2 (0.9)	*p* = 0.08
5. Senior staff in the psychiatric ED encourages open communication between the medical and nursing staff	4.1 (0.8)	4.3 (0.8)	*p* = 0.07
6. Medical and nursing staff in the psychiatric ED encourage cooperation to optimize patient care	4.2 (0.8)	4.3 (0.8)	*p* = 0.13
7. Medical and nursing staff in the psychiatric ED have enough time to perform their work, and also to engage in quality improvement projects	3.4 (1.0)	3.7 (1.1)	*p* = 0.03
			*t* = −2.1

Bold values indicate the overall construct, representing the aggregated measure across all related items.

The second construct, Context, comprises seven statements regarding receptivity to quality improvement initiatives within the ED, based on leaders' attitudes toward change, the decision-making process, and the communication and collaboration between medical and nursing teams. High agreement with the overall Context construct was found, with no significant differences between the pre-implementation phase (4.0, 0.7) and the telepsychiatry phase (4.1, 0.7). One of the items with the lowest agreement was the item about the ED staff having sufficient time for duties and quality improvement projects. For this item, a significant increase was found from the pre-implementation phase (3.4, 1.0) to the telepsychiatry phase (3.7, 1.1; *p* = 0.03) (Table 1).

A multiple regression with Robust Standard Errors was performed to predict overall perceptions of the Evidence construct. Significant predictors of a higher Evidence score included telepsychiatry phase (vs. pre-implementation period; *β* = 0.2, *p* < 0.001), psychiatrist (vs. nurses; *β* = −0.3, *p* < 0.001), and management role (vs. non-management; *β* = 0.2, *p* < 0.01), collectively explaining 23% of the variance. These findings suggest that perceptions about the strength of the evidence for the intervention improved after the implementation of telepsychiatry in the ED, and were higher among managers and among psychiatrists. Seniority in the ED did not significantly affected perceptions on the Evidence construct (Table 2).

**Table 2:** Multiple regression with robust standard errors to predict overall perceptions of overall evidence construct.

Predictors	B	Robust SE (B)	β
Research phase (pre-implementation/ telepsychiatry)	0.5	0.1	0.2***
Profession (psychiatry/ nursing)	−0.7	0.2	−0.3***
Management role (no/ yes)	0.5	0.2	0.2**
Seniority (years of experience working at the ED)	−0.006	0.007	−0.1
*F*		22.51	
*R* ^2^		0.23	
Adjusted *R*^2^		0.22	

** *p* < 0.01.

*** *p* < 0.001.

A multiple regression with Robust Standard Errors was performed to predict overall perceptions of the Context construct. Significant predictors of a higher Context score included telepsychiatry phase (vs. pre-intervention; *β* = 0.2, *p* = 0.02), psychiatrists (vs. nurses; *β* = −0.2, *p* = 0.004), and management role (vs. non-management; *β* = 0.3, *p* < 0.001), collectively explaining 14% of the variance. These findings suggest that perceptions about how supportive the environment was for implementing change improved after the implementation of telepsychiatry in the ED, and were higher among managers, while nurses had lower agreement with the construct. Seniority in the ED did not significantly affect perceptions on the Context construct (Table 3).

**Table 3:** Multiple regression with robust standard errors to predict overall perceptions of overall context construct.

Predictors	B	Robust SE (B)	β
Research phase (pre-implementation/ telepsychiatry)	0.2	0.1	0.1*
Profession (psychiatry/ nursing)	−0.3	0.1	−0.2*
Management role (no/ yes)	0.4	0.1	0.3***
Seniority (years of experience working at the ED)	−0.008	0.006	−0.1
*F*		11.18	
*R* ^2^		0.14	
Adjusted *R*^2^		0.12	

* *p* < 0.05.

*** *p* < 0.001.

Respondents were also asked two open-ended questions: how they believe telepsychiatry will fit patients, and what challenges exist in using telepsychiatry in the ED for involuntary commitment. Using exploratory content analysis, their answers were categorized into CFIR (Consolidated Framework for Implementation Science Research) domains and sub-domains, including implementation process (of telepsychiatry in the ED), inner setting (of the ED), outer setting (i.e., laws, the health system), the characteristics of the intervention (i.e., telepsychiatry), and the characteristics of individuals (e.g., patients, ED staff) (Table 4).

**Table 4:** Perceptions of telepsychiatry's fit and challenges coded into cFIR constructs, compared between pre-implementation (*n* = 118) and implementation (n = 105) phases.

Domain and sub-domains (more than one answer possible)	Definition	No., % of quotes in pre-TP phase (*n* = 287)	No., % of quotes in TP phase (*n* = 205)	Quote of facilitators and barriers
*Implementation process*		*2, 1%*	*0, 0%*	
Reflecting & evaluating	Collect and discuss data on TP's implementation success	2, 1%	0, 0%	“Every change needs a period of time to get used to it” (nurse, management role, do not work at the ED, pre-TP phase, #348)
*Inner setting*		*30, 11%*	*25, 12%*	
Available Resources: space	Quite and private space in the ED to conduct the TP	1, 1%	4, 2%	"Adapted room [for TP]” (nurse, 3 years of experience in ED, pre-TP phase, #324)
				“Finding an intimate and secluded place to conduct the evaluation, without interruptions” (nurse, 26 years of experience in ED, TP phase, #393)
				“There is a need to adapt the therapeutic setting in the ED to different safety requirements” (Attending psychiatrist, management team, TP phase, #508)
Available Resources: Materials & Equipment	ED has equipment (e.g., good internet quality) and available technical team to deliver TP in the ED	29, 10%	21, 10%	"Care must be taken with the quality of the communication-photography, lighting and sound” (Attending psychiatrist, management team, 23 years of experience in ED, pre-TP phase, #17)
				“Technical challenges such as an available network, the integrity of the equipment and the quality of the evaluation execution” (nurse, management team, 20 years of experience in ED, pre-TP phase, #25)
				“The difficulty is technological, the speed and reliability of the connection, the quality of the audio and video” (Attending psychiatrist, management team, 7 years of experience in ED, TP phase, #525)
				“Technical malfunctions, even sometimes on television you don't see the patient's facial expressions properly or body movements, even though this is a very important thing in psychiatric evaluations” (nurse, 2 years of experience in ED, TP phase, #368)
*Innovation characteristics*		*122, 41%*	*71, 35%*	
Innovation evidence-based:	Statements about evidence that support or undermining TP's effectiveness compared with in-person evaluations			
1. General statement based on evidence-based		2, 1%	0	"From years of experience, video evaluation is equivalent to face-to-face evaluation” (Attending psychiatrist, management team, 23 years of experience in ED, pre-TP phase, #17)
				“Today, video is an accepted form of communication” (Attending psychiatrist, management team, 31 years of experience in ED, pre-TP phase, #323)
2. Clinical outcomes based on evidence-based	Including the availability of psychiatric evaluation for the patient and for consultant between the resident and the attending psychiatrist	0, 0%	2, 1%	“So far, the use of TP has accelerated the treatment of ED patients, faster arrival of important decisions such as sedation treatment, issuance of an urgent forced hospitalization order, hospitalization and discharge” (Resident psychiatrist, 4 years of experience in ED, TP phase, #536)
3. Professionalism based on evidence-based	medical professionals promise to meet shared standards of skill and ethics in their work	0, 0%	2, 1%	“The therapist-patient relationship is damaged” (Attending psychiatrist, management team, 14 years of experience in ED, TP phase, #436)
C. Innovation relative advantage:	Statements about support or undermining TP's effectiveness compared with in-person evaluations			
1. General statement		5, 1%	3, 1%	“Optimally, this video is effective and evaluates can be done as in many cases in the community” (Attending psychiatrist, management team, 5 years of experience in ED, pre-TP phase, #14)
				“There is no significant difference between the conclusions from video and in-person evaluations” (Attending psychiatrist, management team, 29 years of experience in ED, pre-TP phase, #329)
				“A video evaluation is suitable for examining 99% of patients” (Attending psychiatrist, management team, 30 years of experience in ED, pre-TP phase, #442)
2. Inner setting outcomes	Including TP's effect on ED length of stay or privacy and confidentiality issues	31, 11%	15, 7%	“The attending psychiatrist that is located at his home will evaluate the subject on video, a long wait in the ED for his arrival will be avoided” (Attending psychiatrist, management team, 8 years of experience in ED, TP phase, #10)
				“Will shorten waiting times during which unusual events [violence] may occur” (Resident psychiatrist, 3 years of experience in ED, pre-TP phase, #84)
				“The existing technology allows an evaluation at the same level and clinical quality as in-person evaluation, and since it significantly shortens the waiting times-it is preferable” (Attending psychiatrist, management team, 8 years of experience in ED, TP phase, #459)
				“Reductions in waiting time for the arrival of an attending psychiatrist in the evening and at night shifts” (ED nurse, management team, 8 years of experience in ED, TP phase, #374)
3. Clinical outcomes	Including the prevalence of violent incidents, high-quality and in-depth evaluations, availability of experts, solutions for patients leaving the ED before evaluation, and data collection from family members	58, 20%	33, 16%	"I don't think it [TP] will fit. I think it is impossible to understand patients and their mental state without seeing them face-to-face (Resident psychiatrist, 2 years of experience in ED, pre-TP phase, #54)
				“I fear that when a patient sees that the staff is using video, it will increase the delusional thoughts” (nurse, management team, 20 years of experience in ED, pre-TP phase, #25)
				“It will be difficult to notice subtle nuances in the evaluation” (Attending psychiatrist, management team, 20 years of experience in ED, pre-TP phase, #282)
				“[Using TP can] will give the patient a feeling that he and his distress are being treated” (ED nurse, pre-TP phase, #286)
				“From a technological point of view, there is no obstacle to producing a good quality evaluation. There is an advantage of the speed with which the evaluation can start” (Resident psychiatrist, 9 years of experience in ED, pre-TP phase, #322)
				“Can be very helpful in cases where the resident psychiatrist has doubts, beyond the court order evaluation” (Resident psychiatrist, 2 years of experience in ED, TP phase, #496)
				“In cases of extreme agitation or complete refusal, it is not possible to conduct an evaluation by TP. Sometimes there are cases that require a frontal evaluation” (Attending psychiatrist, management team, 12 years of experience in ED, pre-TP
				phase, #396)
4. professional team outcomes	Including burnout degree, physical risk from patient violence, and the resident ability to learn from the attending	7, 2%	1, 1%	"Less burnout of attending psychiatrists… The challenge is that the attending at home will turn into the resident in the ED, by evaluating cases that he would not normally check just because the technology makes it possible” (Attending psychiatrist, management team, 20 years of experience in ED, pre-TP phase, #52)
				“The interaction itself is less instructive for the resident psychiatrist” (Resident psychiatrist, 3 years of experience in ED, TP phase, #496)
5. Professionalism	medical professionals promise to meet shared standards of skill and ethics in their work	11, 4%	6, 4%	“The very idea of ​​using video does an injustice to patients when the very traumatic decision of forced hospitalization is made… We should not switch to a remote diagnosis method, certainly regarding forced hospitalization!” (nurse, 23 years of experience in ED, pre-TP phase, #274)
				“It reduces the human encounter a lot” (ED Resident psychiatrist, pre-TP phase, #496)
				“Lack of in-person contact with patients who have difficulties, anyway, will only cause excessive irritation and even exaggerated reactions of the patient. In an in-person meeting, the psychiatrist knows how and what to do to calm the patients” (nurse, management team, do not work at the ED, TP phase, #395)
				“It is difficult to establish contact in an interview that may affect the patient's willingness to share information” (Attending psychiatrist, management team, 5 years of experience in ED, TP phase, #453)
Innovation Adaptability	TP can be modified, tailored, or refined to fit local context or needs of the ED	7, 2%	6, 4%	“A patient is not always willing to stay in the examination room, but is walking around the ED” (nurse, management team, 34 years of experience in ED, pre-TP phase, #28)
				“The problem is that the evaluation can only be performed in one place … there are quite a few cases where the patient is not ready to enter the examination room and this makes it difficult to carry out an evaluation” (nurse, management team, 6 years of experience in ED, TP phase, #365)
*Individual characteristics*		*129, 45%*	*109, 53%*	
Innovation deliverers:	Resident and attending psychiatrists that are delivering TP			
1. Trained and cooperative team		11, 4%	5, 2%	“There is a need for the ED team's support in order to make sure that things are indeed working correctly, and that there are no things that are inaccessible to the attending, for example, behavior in the waiting room, accompanying family members and their attitude towards the patient, etc.” (Resident psychiatrist, one year of experience in ED, pre-TP phase, #40)
				“There is a need for a cooperation and skills for the attending on the video and the resident at the ED” (Attending psychiatrist, management team, 20 years of experience in ED, pre-TP phase, #49)
				“The presence of a resident psychiatrist and a nurse usually completes the picture, and this does not pose a great challenge to the issuance of a court order for involuntary commitment” (Attending psychiatrist, 8 years of experience in ED, TP phase, #469)
				“The combination of a video call with the resident's evaluation is efficient and appropriate” (Attending psychiatrist, 25 years of experience in ED, TP phase, #471)
2. Other aspects related to the team, e.g., providing detailed explanation about TP to patients, experts should consider when TP is suitable, or staff's technological literacy		6, 2%	5, 2%	“It is possible to create a pleasant atmosphere and maintain comfort and privacy and confidentiality through informing patients about this method” (Resident psychiatrist, one year of experience in ED, pre-TP phase, #262)
				“In most cases [TP is doable], but each case should still be considered individually and, if necessary, a frontal examination should be performed” (Attending psychiatrist, management team, 7 years of experience in ED, pre-TP phase, #79)
				“Preparation of the patient and information for the attending is requested” (Attending psychiatrist, management team, 25 years of experience in ED, TP phase, #506)
				“In cases where clinical intervention is needed for a turbulent patient, there can be an advantage to a frontal presence. This can be at the discretion of the resident psychiatrist, whether to ask the attending to arrive physically” (Attending psychiatrist, management team, 8 years of experience in ED, TP phase, #459)
Innovation recipients, and the degree in which TP is suitable based on:	Patients who arrive at the ED and are evaluated by an attending psychiatrist			
1. Patient Mental States and Behavioral Characteristics	Patients' cooperation, their agitated, suspicious state, paranoia, etc.	95, 33%	86, 43%	“[TP] Will only fit calm patients, there will be a problem with violent ones who won't cooperate and won't establish a conversation” (nurse, 8 years of experience in ED, pre-TP phase, #91)
				“It [TP] will not be possible to complete a full examination for an agitated patient” (Resident psychiatrist, one year of experience in ED, pre-TP phase, #317)
				“[the challenge is when] patients refuse to cooperate and does not answer the attending's questions” (Resident psychiatrist, 3 years of experience in ED, TP phase, #483)
				“TP is suitable for all patients, with the exception of those whose mental state is turbulent and does not allow entry into the examination room” (Attending psychiatrist, 8 years of experience in ED, TP phase, #513)
2. patient consent to TP, communication, and preferences		17, 6%	13, 6%	“To get informed consent on the type of evaluation when the patient is in court order status” (Attending psychiatrist, management team, 6 years of experience in ED, pre-TP phase, #44)
				“Will be suitable mainly for patients who do not need translation [to their native language]” (Attending psychiatrist, management team, 15 years of experience in ED, pre-TP phase, #4)
				“TP is suitable for anyone who agrees to be evaluated remotely” (Attending psychiatrist, management team, 34 years of experience in ED, TP phase, #373)
				“Usually this method is suitable and very good in terms of time, but obviously there are exceptions, some people would prefer a frontal evaluation” (nurse, 2 years of experience in ED, TP phase, #368)
*Outer setting*		*4, 2%*	*0, 0%*	
Local Attitudes	Families and patients' attitudes about TP	1, 1%	0	“The patients will tell their families that the evaluation was not done seriously or thoroughly” (Attending psychiatrist, management team, 5 years of experience in ED, pre-TP phase, #352)
Policies & Laws		3, 1%	0	“In the current system structure, including legal issues … forced hospitalization cannot be based on a video call” (Attending psychiatrist, management team, pre-TP phase, #60)

In the pre-implementation phase, the most frequently coded CFIR construct was individual characteristics (129, 45% of all codes found in the pre-implementation phase). This is not surprising, given that a direct question asked how they believe telepsychiatry would suit patients.‏ Most comments addressed the patient's mental state (95, 33%), highlighting anticipated difficulties in conducting telepsychiatry with paranoid or agitated patients. Another highly coded construct was innovation characteristics (122, 41%). These comments commonly suggested an expectation that telepsychiatry would shorten length of stay in the ED. Additionally, there were mostly negative comments (27/30) regarding the ability of attending psychiatrists to conduct a full in-depth evaluation using telepsychiatry, while there were positive comments (11, 4%) about the expectation that telepsychiatry would help resident psychiatrists to more easily communicate with attending physicians and therefore to expedite evaluation for patients. Another important sub-category included statements about potential harm to professionalism from using telepsychiatry (11, 4%). Professionalism is the commitment of medical professionals to uphold shared standards of competence, ethics, and conduct in their work (47). Concerns related to professionalism were raised by both nursing and psychiatry staff, particularly regarding the perceived reduction in the quality of the human interaction. Participants noted challenges in establishing rapport, increased patient distress, and reduced willingness of patients to share information during remote evaluations. There were only 30 comments for the inner setting construct (11%), mainly concerning technical challenges like equipment or internet quality. The least coded CFIR constructs in the pre-implementation phase were implementation process (2, 1%) and outer setting (4, 2%).

In the telepsychiatry phase, the most frequently coded CFIR construct was individual characteristics (109, 53%). The main sub-category identified was the patient's mental state (86, 43%), primarily addressing patients' cooperation or the process of gaining their cooperation during the telepsychiatry evaluation (33, 16%). Another sub-category was the difficulty in conducting telepsychiatry evaluations with agitated or violent patients (29, 14%). There were also many comments about innovation characteristics (71, 35%). The main sub-category was whether an attending psychiatrist can conduct an in-depth psychiatric evaluation using telepsychiatry (18, 9%; with 12 negative comments and 6 positive). 14 (7%) quotes indicated that telepsychiatry shortens the length of stay in the ED. Similar to the pre-implementation phase, positive codes highlighted the convenience of telepsychiatry compared to in-person evaluation (12, 6%). Only 6 (4%) statements addressed issues with professionalism, with 4 (1%) of them mentioning potential harm to professionalism from using TP. The inner setting construct appeared 25 (12%) times and, similar to the pre-implementation phase, included technical challenges associated with telepsychiatry such as the quality of the internet connection. There were no comments about the implementation process and outer setting constructs.

Respondents' answers to the open-ended questions were coded into two categories related to their general perceptions of telepsychiatry: negative responses (e.g., concerns about the validity of evaluations, potential harm to patients or the therapeutic relationship) and positive responses (e.g., improved patient flow in the ED, more timely evaluation and treatment). In the pre-implementation phase, 49 (49%) respondents had negative responses, and 52 (51%) were positive. In the telepsychiatry phase, only 28 (30%) respondents had negative responses, and 66 (70%) were positive, representing a statistically significant change [*χ*^2^_(1,N=195)_ = 7.1,*p* = 0.006].

The answers were also coded related to the fit telepsychiatry to the majority of the patients. In the pre-implementation phase, only 39 respondents mentioned their opinion on the degree telepsychiatry fits to patients. Of them, 28 (72%) thought telepsychiatry would fit patients arriving to the ED, and 11 (28%) thought it would not fit most of the participants. In the telepsychiatry phase, 57 respondents mentioned their opinion on that topic. Of them, 47 (83%) thought telepsychiatry appropriate for the majority of the patients, while 10 (17%) thought it did not fit the majority of the patients. This increase in support for telepsychiatry as a method which fits the majority of patients was not statistically significant.

## 4 Discussion

We evaluated changes in implementers' views of innovations, specifically examining the implementation process of telepsychiatry in the ED setting and how these views evolved over time. We investigated the perceptions of nurses and psychiatrists working in the ED regarding the use of telepsychiatry as part of the evaluation process for determining the need for involuntary psychiatric hospitalization. A questionnaire assessed perceptions using two constructs: Evidence-assessing the strength, feasibility, and preference for telepsychiatry over face-to-face evaluations; and Context-assessing receptivity to quality improvement initiatives based on leaders' attitudes toward change, decision-making processes, and communication and collaboration between teams. Scores on both constructs were generally high. Evidence scores increased significantly from the pre-implementation phase to the telepsychiatry phase. Context scores did not change significantly, although there was a significant increase in scores on one item about ED staff having sufficient time for duties. A regression model identified the telepsychiatry phase, psychiatrists, and management role as significant factors influencing perceptions of both Evidence and Context.‏ Respondents' views on telepsychiatry's fit for patients and challenges in the ED were categorized into CFIR domains. In both phases, many comments mentioned individual and innovation characteristics, with pre-implementation concerns about patient mental state and evaluation feasibility, and telepsychiatry phase concerns about patient cooperation and agitated patients. A few concerns about the impact of telepsychiatry on professionalism arose in both phases. Overall, negative responses decreased significantly during the telepsychiatry phase, while perceptions of telepsychiatry's fit for patients increased but not statistically significantly.

In implementing any innovation or change, teams often need time to adapt and integrate the new method into their routine (48, 49). Accordingly, we observed a significant increase in respondents' perceptions during the telepsychiatry phase regarding its acceptance, feasibility, and the belief that its advantages outweigh the disadvantages. Interestingly, nurses' perceptions of telepsychiatry were less positive compared to those of the psychiatrists. During the telepsychiatry phase, telepsychiatry was primarily operated by resident psychiatrists from the ED, and attending psychiatrists, most of whom held management roles and had positive perceptions of telepsychiatry. Nurses were generally not involved in the telepsychiatry preparations and often were not present during the evaluations. This lack of engagement may have influenced their perceptions, leading to less positive statements regarding telepsychiatry's acceptability, feasibility, and advantages. When implementing innovations in EDs, especially technological ones, it is crucial to consider the perceptions of all implementers, particularly nurses, for successful implementation (49–51). In the current study, it was observed that nurses played a less central role in the implementation of telepsychiatry in the ED, which likely contributed to their differing perspectives on telepsychiatry compared to psychiatrists. While additional orientation time might have helped, the distinct roles and responsibilities of nurses and psychiatrists naturally influence their viewpoints regarding these innovations.

One factor to consider in the success of innovation is the degree to which it fits to users and recipients (41). In our study, three groups were relevant: resident psychiatrists, attending psychiatrists, and patients. Our findings suggest that both resident and attending psychiatrists were able to accept and use telepsychiatry with minor issues, such as technical equipment problems and the need for additional training, which improved during the telepsychiatry phase. The findings in this study were about whether telepsychiatry is suitable for all patients. Respondents expressed concerns about telepsychiatry's applicability to various mental states and behavioral characteristics, particularly with agitated, paranoid, or uncooperative patients. Interestingly, after using telepsychiatry, their perceptions regarding telepsychiatry's applicability to most patients improved, and they expressed fewer concerns about this issue compared to the pre-implementation phase. Our findings may help to assuage doubts that are often expressed regarding this issue (52, 53).

We saw relatively few comments (4%) expressing concerns about professionalism, but they were noteworthy, because we had not directly asked about this topic. Medical professionalism is a belief system where professionals commit to shared competency standards and ethical values, promising to uphold these in their work and ensuring the public and patients can expect high standards of care (47). The concerns raised focused on whether telepsychiatry allows psychiatrists to establish a meaningful therapeutic relationship, secure patient cooperation, and convey a sense of care. Ethical aspects of telepsychiatry have likewise been discussed in the literature (54–56). Consistently, in our recent study of 36 psychiatrists examining telepsychiatry for involuntary admissions, professionalism concerns also emerged. Although psychiatrists acknowledged its efficiency, views diverged regarding its professional adequacy: some considered telepsychiatry a sufficient alternative to in-person evaluation, whereas others emphasized the stronger therapeutic connection enabled by face-to-face encounters (57). These concerns highlight an important issue for clinicians and policymakers. Accordingly, telepsychiatry implementation should be accompanied by rigorous evaluation of its impact not only on patient outcomes but also on the ability to establish a meaningful patient connection.

Respondents rarely discussed the implementation process and outer setting constructs before implementing telepsychiatry, and even less often during the telepsychiatry phase. This likely stems from these factors being less immediately relevant to the nurses' and psychiatrists' day-to-day clinical experiences and direct interactions with telepsychiatry. Clinical teams in the ED, dealing with an intensive environment (9–11), may have focused more on practical, immediate concerns such as technology usability and patient cooperation, rather than broader organizational or external factors influencing implementation-despite the known importance of these factors in determining the success of innovations. Our findings in this regard do echo those of other studies. For example, Kathryn Alison and colleagues (2024) explored ED nurses' perceptions of telemedicine use for sexual assault exams and its implementation influences. Through semi-structured interviews with 15 nurses from 13 EDs, they identified facilitators and barriers, noting that none of the responses related to the implementation process domain (58). Another key component of the implementation process is the training conducted prior to telepsychiatry implementation, along with the designation of a lead attending psychiatrist and a resident psychiatrist at each site, who served as innovation champions. In our study, these elements were not mentioned by participants, which may reflect the unique characteristics and demands of the ED setting rather than a lack of importance of these factors for successful implementation. Indeed, both training and the presence of innovation champions have been identified as important facilitators of successful telepsychiatry implementation (57, 59, 60).

This study has several limitations. Relying solely on the perspectives of clinical and management teams to understand the implementation process of telepsychiatry in the ED does not provide a comprehensive picture. However, the sample size in both phases was relatively large and offered unique insights from eight different settings. The use of a questionnaire to explore respondents' perceptions also presents a limitation, as it lacks the ability to explore deeper into participants' answers. Nonetheless, we utilized a validated questionnaire that included both close-ended and open-ended questions to capture a broad range of respondents' perceptions as thoroughly as possible. Additionally, this study evaluated participants' perceptions of telepsychiatry after only a few months of use, while their views may change as they become more accustomed to the technology. To the best of our knowledge, this is the first study to investigate the implementation of telepsychiatry in the ED for involuntary hospitalization, specifically examining the perspectives of both nurses and psychiatrists. Future longitudinal studies can track changes in perceptions over a more extended period. Conducting in-depth interviews with a broader range of stakeholders, including policymakers and patients, alongside clinical and management teams, can provide a more comprehensive understanding of the implementation of telepsychiatry in the ED for involuntary hospitalization.

## 5 Conclusions

In this paper, we examined changes in implementers' perceptions before and after the implementation of an innovation. We evaluated the perceptions of nurses and psychiatrists working in the ED regarding the implementation of telepsychiatry for involuntary hospitalization. We utilized an online questionnaire, focusing on two constructs: Evidence and Context. We found that during the telepsychiatry phase, psychiatrists and managers had higher perceptions of both the Evidence and Context constructs. In the pre-implementation phase, respondents' comments focused on patients' mental state and the feasibility of evaluations, while in the telepsychiatry phase, issues related to patient cooperation and agitation were emphasized. In both phases, comments highlighted individual and innovation characteristics as important. This study identified several facilitators associated with changes in implementers' perceptions, among which sustained use of the innovation over time appears to play a role. As implementers become more familiar with the innovation, they may develop more positive perceptions and recognize its potential benefits.

## Data Availability

The raw data supporting the conclusions of this article will be made available by the authors, without undue reservation.
